# Determination of the Effective Parameters for Estimating the Temperature Mode at Braking

**DOI:** 10.3390/ma19122611

**Published:** 2026-06-17

**Authors:** Aleksander Yevtushenko

**Affiliations:** Faculty of Mechanical Engineering, Bialystok University of Technology, 45C Wiejska Street, 15-351 Bialystok, Poland; a.yevtushenko@pb.edu.pl

**Keywords:** braking, friction heating, temperature mode, heat accumulation, thermal surface layer

## Abstract

A methodology for determining two parameters was proposed: the effective depth of heat penetration, and the thickness of the surface layer accumulating a given amount of heat. Explicit formulas allowing estimation of the values of these parameters for a semi-infinite body heated by heat flux with a variable time profile of intensity were obtained. Ten time profiles corresponding to different types of braking were analysed. The obtained results can be used at the design stage to determine the temperature mode and then to select materials for the friction elements of a disc braking system.

## 1. Introduction

Determining the temperature mode of friction pair elements is essential for assessing the effectiveness and reliability of their materials under given operating conditions [1,2]. Temperature significantly determines the friction and wear characteristics of materials [3,4,5], as well as their structural transformations [6,7] and the intensity of physical, chemical and mechanical processes occurring in frictional contact [8,9]. Reliable, experimental determination of the temperature field, especially the temperature of the friction surface on natural objects, involves in most cases significant technical difficulties as well as material and financial costs [10]. Therefore, the development of analytical models for determining the spatial–temporal temperature distribution of friction pair elements is of particular interest.

Initially, the friction elements do not heat up throughout their thickness. Most often, heat generated by friction accumulates in the surface thermal layer, where a rapid temperature change occurs [11,12]. Furthermore, the temperature increase in such a layer at a given distance from the friction surface becomes negligible compared to the temperature increase on that surface.

When determining the temperature mode of braking systems, an important input parameter is the effective depth of heat penetration into each element of the friction pair (e.g., pad and disc) [13,14,15]. The accuracy of solutions to thermal problems of friction during braking depends on correct estimation of this parameter [16]. Typically, this parameter is the thickness of the surface layer, in which the vast majority of heat is concentrated at a given moment of the braking process. This parameter is determined in the following form [17,18,19]:

(1) $$\delta =\lambda \sqrt{kt_{s}},$$

where k is the thermal diffusivity of the material, and $t_{s}$ is the stopping time. The coefficient λ in Formula (1) takes different values depending on the design requirements. Thus, in the case of short-term braking at δ<d, where d is the thickness of the friction element, the values $\lambda =\sqrt{3}≅1.73$ [20,21] or λ=1.75 [22] are used. This value λ=3.2 is used to determine the maximum thickness of the layer at which the effect of temperature on the change of mechanical properties should be taken into account [23].

Therefore, the choice of the coefficient λ in Formula (1) in each specific case depends on the physical justification underlying the concept of thermal layer thickness. In engineering practice, it can be associated with recording rapid temperature changes in the surface layer of the body or with heat accumulation in a layer of thickness δ. The aim of this article is to develop a general methodology for determining the coefficient λ in Formula (1) based on analytical solutions of initial-boundary thermal conductivity problems for a semi-space heated by a heat flux with a variable temporal profile of intensity.

## 2. Problem Formulation

The intensive braking process in most modern machines is transient. Therefore, when formulating the thermal problem of friction, convective heat exchange between the elements of the friction pair and the surrounding environment can be neglected [16]. Specifically, it has been demonstrated that for standard heat transfer coefficient values ranging from 20 to 60 W m^−2^ K ^−1^, in the absence of forced cooling and for Fourier numbers less than 10, this ambient heat exchange has a negligible effect on calculations of temperature during braking. Additionally, it is assumed that the vast majority of heat propagates inward toward each element along the normal to the friction surface, meaning that temperature field distributions can be determined using one-dimensional parabolic heat conduction equations with appropriate initial and boundary conditions. Moreover, the thermophysical properties of the materials are assumed to remain unchanged during the heating process.

The intensity of the heat flux generated on the friction surface of a braking system component changes over time in various ways. A classification of the temporal profiles of heat flux intensity during braking was presented in [24,25], including the following time profiles:

(2) $$q_{i}(t)=q_{0}q_{i}^{*}(t),\;0\leq \;t\leq t_{s},\;i=1,2,\ldots ,10,$$

(3) $$q_{0}=\gamma fp_{0}V_{0},$$

(4) $$q_{1}^{*}(t)=2(1-t^{*}),\;q_{2}^{*}(t)=2t^{*},\;q_{3}^{*}(t)=1.5\sqrt{1-t^{*}},\;q_{4}^{*}(t)=1.5\sqrt{t^{*}},$$

(5) $$q_{5}^{*}(t)=3{\left(1-t^{*}\right)}^{2},\;q_{6}^{*}(t)=3{\left(t^{*}\right)}^{2},\;q_{7}^{*}(t)=6t^{*}(1-t^{*}),\;q_{8}^{*}(t)=1.2(1-t^{*})(1+2t^{*}),$$

(6) $$q_{9}^{*}(t)=2.4t^{*}(1.5-t^{*}),\;q_{10}^{*}(t)=6\sqrt{t^{*}}(1-\sqrt{t^{*}}),\;t^{*}=tt_{s}^{-1},$$

where γ is the heat partition rate [26,27], $f_{0}$ is the coefficient of friction at initial temperature, $p_{0}$ is the nominal value of the contact pressure, and $V_{0}$ is the initial velocity. It should be noted that the total specific friction work performed by the friction pair elements during braking at each of the profiles (2)–(6) is the same, and equal to:

(7) $$\int_{0}^{t_{s}}q_{i}(s)ds=q_{0}t_{s},\;i=1,2,\ldots ,10,$$

which enables further comparative analysis of the obtained results.

With such assumptions, the following initial-boundary thermal conductivity problem for the semi-space z≥0 was selected as the model problem to describe the space-time distribution of temperature initiated by short-term frictional heating during braking:

(8) $$\frac{\partial^{2}T_{i}(z,t)}{\partial z^{2}}=\frac{1}{k}\frac{\partial T_{i}(z,t)}{\partial t},\;z>0,\;\;0<\;t\leq t_{s},$$

(9) $${\left.K\frac{\partial T_{i}(z,t)}{\partial z}\right|}_{z=0}=-q_{i}(t),\;\;\;0<\;t\leq t_{s},$$

(10) $$T_{i}(z,t)\to T_{0},\;z\to \infty ,\;\;\;0<t\leq t_{s},$$

(11) $$T_{i}(z,0)=T_{0},$$

where $T_{i}(z,t)$ is the temperature field corresponding to the temporal profile $q_{i}(t)$, i=1,2,…,10, $T_{0}$ is the initial temperature, and *K* is the coefficient of thermal conductivity.

The solutions to the problems (8)–(11) were presented in article [24]. On the basis of these solutions, the temperature rise $\Theta_{i}(z,t)=T_{i}(z,t)-T_{0}$, i=1,2,…,10 at the stop moment $t=t_{s}$ was written in the form:

(12) $$\Theta_{i}(z,t_{s})=\frac{2q_{0}\sqrt{kt_{s}}}{K}\Theta_{i}^{*}(z,t_{s}),\;\;z\geq 0,$$

(13) $$\Theta_{1}^{*}(z,t_{s})=\frac{4}{3}\left[\left(1-2Z^{2}\right)\mathrm{ierfc}\;Z+Z\;\mathrm{erfc}\;Z\right],$$

(14) $$\Theta_{2}^{*}(z,t_{s})=\frac{4}{3}\left[2\left(1+Z^{2}\right)\mathrm{ierfc}\;Z\;-Z\;\mathrm{erfc}\;Z\right],$$

(15) $$\begin{array}{l} \Theta_{3}^{*}(z,t_{s})=\left(\frac{12}{7}-\frac{493}{280}Z^{2}-\frac{17}{70}Z^{4}-\frac{1}{70}Z^{6}\right)\mathrm{ierfc}\;Z+\\ \;\;\;\;\;\;\;\;\;\;\;\;\;\;\;\;\;\;\;\;\;+Z\left(\frac{87}{112}+\frac{4}{35}Z^{2}+\frac{1}{140}Z^{4}\right)\mathrm{erfc}\;Z, \end{array}$$

(16) $$\Theta_{4}^{*}(z,t_{s})=\frac{3}{4}\sqrt{\pi }\left(\mathrm{erfc}\;Z-2Z\;\mathrm{ierfc}\;Z\right),$$

(17) $$\Theta_{5}^{*}(z,t_{s})=\frac{2}{5}\left[\left(3-2Z^{2}+4Z^{4}\right)\mathrm{ierfc}\;Z+Z\left(3-2Z^{2}\right)\mathrm{erfc}\;Z\right],$$

(18) $$\Theta_{6}^{*}(z,t_{s})=\frac{2}{5}\left[\left(8+18Z^{2}+4Z^{4}\right)\mathrm{ierfc}\;Z-Z\left(7+2Z^{2}\right)\mathrm{erfc}\;Z\right],$$

(19) $$\Theta_{7}^{*}(z,t_{s})=\frac{8}{5}\left[\left(1-4Z^{2}-2Z^{4}\right)\mathrm{ierfc}\;Z+Z\left(1+Z^{2}\right)\mathrm{erfc}\;Z\right],$$

(20) $$\Theta_{8}^{*}(z,t_{s})=\frac{4}{25}\left[\left(9-26Z^{2}-8Z^{4}\right)\mathrm{ierfc}\;Z+Z\left(9+4Z^{2}\right)\mathrm{erfc}\;Z\right],$$

(21) $$\Theta_{9}^{*}(z,t_{s})=\frac{4}{25}\left[\left(14-6Z^{2}-8Z^{4}\right)\mathrm{ierfc}\;Z-Z\left(1-4Z^{2}\right)\mathrm{erfc}\;Z\right],$$

(22) $$\Theta_{10}^{*}(z,t_{s})=\left(3\sqrt{\pi }+4Z\right)\mathrm{erfc}\;Z-\left(8+6\sqrt{\pi }Z+8Z^{2}\right)\mathrm{ierfc}\;Z,$$

(23) $$\mathrm{ierfc}\;Z=\frac{1}{\sqrt{\pi }}e^{-Z^{2}}-Z\;\mathrm{erfc}\;Z,\;\;\mathrm{erfc}\;Z=1-\mathrm{erf}\;Z,\;\;\mathrm{erf}\;Z=\frac{2}{\sqrt{\pi }}\int_{0}^{Z}e^{-x^{2}}dx\;,$$

(24) $$Z=\frac{z}{2\sqrt{kt_{s}}}.$$

## 3. Effective Depth of Heat Penetration

The effective depth of heat penetration was defined as the distance from the surface of friction at which the temperature differs by a given amount from the surface temperature. According to this definition, this distance was estimated using the following parameter:

(25) $$\Theta_{\delta ,i}^{*}\equiv \frac{{\hat{\Theta }}_{\delta ,i}^{*}}{{\hat{\Theta }}_{i}^{*}},\;i=1,2,\ldots ,10,$$

where ${\hat{\Theta }}_{\delta ,i}^{*}\equiv \Theta_{i}^{*}(\delta ,t_{s})$, ${\hat{\Theta }}_{i}^{*}\equiv \Theta_{i}^{*}(0,t_{s})$.

It should be noted that the dimensionless variable *Z* (24) at z=δ is equal to:

(26) $${\left.Z\right|}_{z=\delta }=0.5\lambda \equiv \Lambda .$$

Next, the dimensionless temperature rises ${\hat{\Theta }}_{\delta ,i}^{*}$ in Formula (25) were determined from Formulas (13)–(23) by formally replacing the dimensionless variable Z (24) on the coefficient Λ (26).

The increases in the temperature ${\hat{\Theta }}_{i}^{*}$ on the heated surface were found by substituting z=0 (Z=0) into Formulas (13)–(24). As a result, the following were obtained:

(27) $${\hat{\Theta }}_{1}^{*}=\frac{4}{3\sqrt{\pi }},\;{\hat{\Theta }}_{2}^{*}=\frac{8}{3\sqrt{\pi }},\;{\hat{\Theta }}_{3}^{*}=\frac{12}{7\sqrt{\pi }},\;{\hat{\Theta }}_{4}^{*}=\frac{3}{4}\sqrt{\pi },\;{\hat{\Theta }}_{5}^{*}=\frac{6}{5\sqrt{\pi }},$$

(28) $${\hat{\Theta }}_{6}^{*}=\frac{16}{5\sqrt{\pi }},\;{\hat{\Theta }}_{7}^{*}=\frac{8}{5\sqrt{\pi }},\;2,\;{\hat{\Theta }}_{9}^{*}=\frac{56}{25\sqrt{\pi }},\;{\hat{\Theta }}_{10}^{*}=\frac{3\pi -8}{\sqrt{\pi }}.$$

Substituting the temperature rises ${\hat{\Theta }}_{\delta ,i}^{*}$ and ${\hat{\Theta }}_{i}^{*}$ determined in this way into Formula (25) yields:

(29) $$\Theta_{\delta ,1}^{*}=\sqrt{\pi }\left[\left(1-2\Lambda^{2}\right)\mathrm{ierfc}\;\Lambda +\Lambda \;\;\mathrm{erfc}\;\Lambda \right],$$

(30) $$\Theta_{\delta ,2}^{*}=\sqrt{\pi }\left[\left(1+\Lambda^{2}\right)\mathrm{ierfc}\;\Lambda -\frac{1}{2}\Lambda \;\;\mathrm{erfc}\;\Lambda \right],$$

(31) $$\begin{array}{l} \Theta_{\delta ,3}^{*}=\sqrt{\pi }\left[\left(1-\frac{493}{480}\Lambda^{2}-\frac{17}{120}\Lambda^{4}-\frac{1}{120}\Lambda^{6}\right)\mathrm{ierfc}\;\Lambda +\right.\\ \;\;\;\;\;\;\;\;\;\;\;\;\;\;\;\;\;\;\;\;\;\;\;+\left.\Lambda \left(\frac{203}{448}+\frac{1}{15}\Lambda^{2}+\frac{1}{240}\Lambda^{4}\right)\mathrm{erfc}\;\Lambda \right], \end{array}$$

(32) $$\Theta_{\delta ,4}^{*}=\mathrm{erfc}\;\Lambda -2\Lambda \;\mathrm{ierfc}\;\Lambda ,$$

(33) $$\Theta_{\delta ,5}^{*}=\sqrt{\pi }\left[\left(1-\frac{2}{3}\Lambda^{2}+\frac{4}{3}\Lambda^{4}\right)\mathrm{ierfc}\;\;\Lambda +\Lambda \left(1-\frac{2}{3}\Lambda^{2}\right)\mathrm{erfc}\;\Lambda \right],$$

(34) $$\Theta_{\delta ,6}^{*}=\sqrt{\pi }\left[\left(1+\frac{9}{4}\Lambda^{2}+\frac{1}{2}\Lambda^{4}\right)\mathrm{ierfc}\;\Lambda -\Lambda \left(\frac{7}{8}+\frac{1}{4}\Lambda^{2}\right)\mathrm{erfc}\;\Lambda \right],$$

(35) $$\Theta_{\delta ,7}^{*}=\sqrt{\pi }\left[\left(1-4\Lambda^{2}-2\Lambda^{4}\right)\mathrm{ierfc}\;\Lambda +\Lambda \left(1+\Lambda^{2}\right)\mathrm{erfc}\;\Lambda \right],$$

(36) $$\Theta_{\delta ,8}^{*}=\sqrt{\pi }\left[\left(1-\frac{26}{9}\Lambda^{2}-\frac{8}{9}\Lambda^{4}\right)\mathrm{ierfc}\;\Lambda +\Lambda \left(1+\frac{4}{9}\Lambda^{2}\right)\mathrm{erfc}\;\Lambda \right],$$

(37) $$\Theta_{\delta ,9}^{*}=\sqrt{\pi }\left[\left(1-\frac{3}{7}\Lambda^{2}-\frac{4}{7}\Lambda^{4}\right)\mathrm{ierfc}\;\Lambda -\Lambda \left(\frac{1}{14}-\frac{2}{7}\Lambda^{2}\right)\mathrm{erfc}\;\Lambda \right],$$

(38) $$\Theta_{\delta ,10}^{*}=\frac{\sqrt{\pi }}{\left(3\pi -8\right)}\left[\left(3\sqrt{\pi }+4\Lambda \right)\mathrm{erfc}\;\Lambda -\left(8+6\sqrt{\pi }\Lambda +8\Lambda^{2}\right)\mathrm{ierfc}\;\Lambda \right].$$

**Figure 1:**
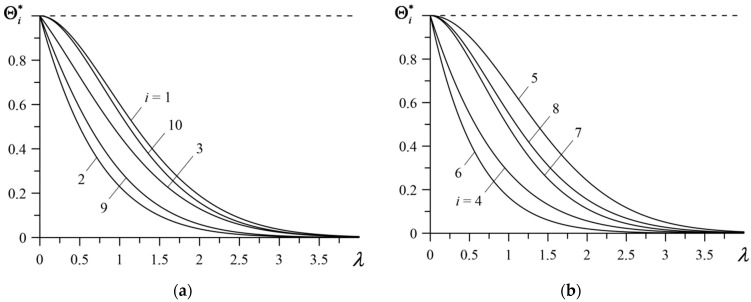
Changes in the parameters $\Theta_{\delta ,i}^{*}$ (25) with increases in the coefficient λ: (**a**) i=1, 2, 3, 9, 10; (**b**) i=4, 5, 6, 7, 8.

From Formulas (29)–(38), it follows that the ratio of the temperature increase at depth z=δ (1) to the temperature increase on the heated surface z=0 depends only on the coefficient λ. The dependencies of parameters $\Theta_{\delta ,i}^{*}$, i=1,2,…,10 (29)–(38) on the coefficient λ are presented in Figure 1.

It can be seen that the values of the parameters $\Theta_{\delta ,i}^{*}$ decrease monotonically from one to zero with increases in the coefficient λ. This decrease $\Theta_{\delta ,i}^{*}$ is a result of the temperature decreasing with distance from the heated surface. At a distance $\delta \geq 3.5\sqrt{kt_{s}}$ from the heated surface, the temperature practically becomes equal to the initial value. The values of the coefficient λ corresponding to the selected values of the parameters $\Theta_{\delta ,i}^{*}$, i=1,2,…,10 values are given in Table 1.

It was established that during braking with constant deceleration (*i* = 1) the effective heat penetration depth at the 5% level ($\Theta_{\delta ,1}^{*}=0.05$) can be estimated using Formula (1) at λ=2.83. However, in the same braking mode the temperature at distance δ (1) is 1% of the surface temperature at λ=3.64. The largest and smallest distances at which the temperature reaches 1% of the temperature of the heated surface occur at *i* = 5 (λ=3.81) and *i* = 6 (λ=2.3), respectively.

## 4. Effective Thickness of the Heat-Accumulating Layer

Another approach for determining the coefficient λ in Formula (1) is based on the concept of heat accumulation in a surface layer of fixed thickness. For this purpose, the following parameter of thermal saturation of the layer of the thickness δ was defined:

(39) $$Q_{\delta ,i}^{*}=\frac{Q_{\delta ,i}}{Q_{i}},\;i=1,2,\ldots ,10,$$

where

(40) $$Q_{\delta ,i}=\rho cA_{a}\int_{0}^{\delta }\Theta_{i}(z,t_{s})dz,\;Q_{i}=\rho cA_{a}\int_{0}^{\infty }\Theta_{i}(z,t_{s})dz,$$

are the amounts of heat absorbed during braking to the layer 0≤z≤δ and semi-space z≥0 (δ→∞), and $\Theta_{i}(z,t_{s})$ are the temperature rises (12), $A_{a}$ is the area of the nominal contact region, c is the specific heat, and ρ is the density of the body material.

Based on Formulas (12) and (40), the thermal saturation parameter (39) was written in the following form:

(41) $$Q_{\delta ,i}^{*}=\frac{{\hat{Q}}_{\delta ,i}}{{\hat{Q}}_{i}},\;i=1,2,\ldots ,10,$$

where

(42) $${\hat{Q}}_{\delta ,i}=\int_{0}^{\delta }\Theta_{i}^{*}(z,t_{s})dz,\;{\hat{Q}}_{i}=\int_{0}^{\infty }\Theta_{i}^{*}(z,t_{s})dz.$$

Taking into account the form of integrand functions $\Theta_{i}^{*}(z,t_{s})$ (13)–(24) in the first of the Formulas (42), the following were obtained:

(43) $${\hat{Q}}_{\delta ,1}=\frac{4}{3}\left(J_{0}-2J_{2}+2I_{3}\right),$$

(44) $${\hat{Q}}_{\delta ,2}=\frac{4}{3}\left(2J_{0}+2J_{2}-3I_{1}-2I_{3}\right),$$

(45) $${\hat{Q}}_{\delta ,3}=\frac{12}{7}J_{0}-\frac{493}{280}J_{2}-\frac{17}{70}J_{4}-\frac{1}{70}J_{6}-\frac{105}{112}I_{1}+\frac{105}{56}I_{3}+\frac{1}{4}I_{5}+\frac{1}{70}I_{7},$$

(46) $${\hat{Q}}_{\delta ,4}=\frac{3}{4}\sqrt{\pi }\left(I_{0}+2I_{2}-2J_{1}\right),$$

(47) $${\hat{Q}}_{\delta ,5}=\frac{2}{5}\left(3J_{0}-2J_{2}+4J_{4}-4I_{5}\right),$$

(48) $${\hat{Q}}_{\delta ,6}=\frac{2}{5}\left(8J_{0}+18J_{2}+4J_{4}-15I_{1}-20I_{3}-4I_{5}\right),$$

(49) $${\hat{Q}}_{\delta ,7}=\frac{4}{5}\left(J_{0}-4J_{2}-2J_{4}+5I_{3}+2I_{5}\right),$$

(50) $${\hat{Q}}_{\delta ,8}=\frac{4}{25}\left(9J_{0}-26J_{2}-8J_{4}+30I_{3}+8I_{5}\right),$$

(51) $${\hat{Q}}_{\delta ,9}=\frac{4}{25}\left(14J_{0}-6J_{2}-8J_{4}-15I_{1}+10I_{3}+8I_{5}\right),$$

(52) $${\hat{Q}}_{\delta ,10}=3\sqrt{\pi }I_{0}+12I_{1}+6\sqrt{\pi }I_{2}+8I_{3}-8J_{0}-6\sqrt{\pi }J_{1}-8J_{2},$$

where

(53) $$J_{n}=\sqrt{kt_{s}}J_{n}^{*},\;n=0,1,2,4,6;\;I_{m}=\sqrt{kt_{s}}I_{m}^{*},\;m=0,1,2,3,5,7,$$

(54) $$J_{n}^{*}=\frac{2}{\sqrt{\pi }}\int_{0}^{\Lambda }Z^{n}e^{-Z^{2}}dZ\;,\;I_{m}^{*}=2\int_{0}^{\Lambda }Z^{m}\mathrm{erfc}\;Z\;dZ,$$

and the parameter Λ was defined by Formula (26). In calculating the definite integrals $J_{n}^{*}$ (54), the results for indefinite integrals 1.3.3.1, 1.3.3.4 and 1.3.3.7 [28] were used. As a result, the following were obtained:

(55) $$J_{0}^{*}=\mathrm{erf}\;\Lambda ,$$

(56) $$J_{1}^{*}=\frac{1}{\sqrt{\pi }}\left(1-e^{-\Lambda^{2}}\right),$$

(57) $$J_{2}^{*}=\frac{1}{2}\mathrm{erf}\;\Lambda -\frac{\Lambda }{\sqrt{\pi }}e^{-\Lambda^{2}},$$

(58) $$J_{4}^{*}=\frac{3}{4}\mathrm{erf}\;\Lambda -\left(\frac{3}{2}+\Lambda^{2}\right)\frac{\Lambda }{\sqrt{\pi }}e^{-\Lambda^{2}},$$

(59) $$J_{6}^{*}=\frac{15}{8}\mathrm{erf}\;\Lambda -\left(\frac{15}{4}+\frac{5}{2}\Lambda^{2}+\Lambda^{4}\right)\frac{\Lambda }{\sqrt{\pi }}e^{-\Lambda^{2}}.$$

Similarly, based on the indefinite integrals 1.51.4 and 1.5.1.5 [29], the following results were obtained:

(60) $$I_{0}^{*}=2\left(\frac{1}{\sqrt{\pi }}-\mathrm{ierfc}\;\Lambda \right),$$

(61) $$I_{1}^{*}=\frac{1}{2}\mathrm{erf}\;\Lambda -\Lambda \mathrm{ierfc}\;\Lambda ,$$

(62) $$I_{2}^{*}=\frac{2}{3}\left[\frac{1}{\sqrt{\pi }}\left(1-e^{-\Lambda^{2}}\right)-\Lambda^{2}\mathrm{ierfc}\;\Lambda \right],$$

(63) $$I_{3}^{*}=\frac{3}{8}\left(\mathrm{erf}\;\Lambda -\frac{4}{3}\Lambda^{3}\mathrm{ierfc}\;\Lambda -\frac{2\Lambda }{\sqrt{\pi }}e^{-\Lambda^{2}}\right),$$

(64) $$I_{5}^{*}=\frac{5}{8}\left[\mathrm{erf}\;\Lambda -\frac{2}{3}\Lambda^{5}\mathrm{ierfc}\;\Lambda -\left(1+\frac{2}{3}\Lambda^{2}\right)\frac{2\Lambda }{\sqrt{\pi }}e^{-\Lambda^{2}}\right],$$

(65) $$I_{7}^{*}=\frac{105}{64}\left[\mathrm{erf}\;\Lambda -\frac{16}{105}\Lambda^{7}\mathrm{ierfc}\;\Lambda -\left(1+\frac{2}{3}\Lambda^{2}+\frac{4}{15}\Lambda^{4}\right)\frac{2\Lambda }{\sqrt{\pi }}e^{-\Lambda^{2}}\right].$$

After substituting integrals (53), (55)–(65) to the right-hand sides of the Formulas (43)–(52), the following was determined:

(66) $${\hat{Q}}_{\delta ,i}=\sqrt{kt_{s}}{\hat{Q}}_{\delta ,i}^{*}\;,\;i=1,2,\ldots ,10,$$

where

(67) $${\hat{Q}}_{\delta ,1}^{*}=\mathrm{erf}\;\Lambda -\frac{4}{3}\Lambda^{3}\mathrm{ierfc}\;\Lambda +\frac{2\Lambda }{3\sqrt{\pi }}e^{-\Lambda^{2}},$$

(68) $${\hat{Q}}_{\delta ,2}^{*}=\mathrm{erf}\;\Lambda +\frac{3}{4}\Lambda \left(3+\Lambda^{2}\right)\mathrm{ierfc}\;\Lambda -\frac{3\Lambda }{8\sqrt{\pi }}e^{-\Lambda^{2}},$$

(69) $$\begin{array}{l} {\hat{Q}}_{\delta ,3}^{*}=\frac{931}{896}\mathrm{erf}\;\Lambda +\Lambda \left(\frac{105}{112}-\frac{105}{112}\Lambda^{2}-\frac{1}{12}\Lambda^{4}-\frac{1}{280}\Lambda^{6}\right)\mathrm{ierfc}\;\Lambda +\\ \;\;\;\;\;\;\;\;\;\;\;\;\;\;\;\;\;\;\;\;\;\;+\left(\frac{185}{448}+\frac{131}{3360}\Lambda^{2}+\frac{1}{560}\Lambda^{4}\right)\frac{\Lambda }{\sqrt{\pi }}e^{-\Lambda^{2}}, \end{array}$$

(70) $${\hat{Q}}_{\delta ,4}^{*}=1-\sqrt{\pi }\left(\frac{3}{2}+\Lambda^{2}\right)\mathrm{ierfc}\;\Lambda +\frac{1}{2}e^{-\Lambda^{2}},$$

(71) $${\hat{Q}}_{\delta ,5}^{*}=\mathrm{erf}\;\Lambda +\frac{8}{15}\Lambda^{5}\mathrm{ierfc}\;\Lambda +\left(1-\frac{2}{3}\Lambda^{2}\right)\frac{2\Lambda }{5\sqrt{\pi }}e^{-\Lambda^{2}},$$

(72) $${\hat{Q}}_{\delta ,6}^{*}=\mathrm{erf}\;\Lambda +2\Lambda \left(3+2\Lambda^{2}+\frac{4}{15}\Lambda^{4}\right)\mathrm{ierfc}\;\Lambda -\left(2+\frac{1}{3}\Lambda^{2}\right)\frac{4\Lambda }{5\sqrt{\pi }}e^{-\Lambda^{2}},$$

(73) $${\hat{Q}}_{\delta ,7}^{*}=\mathrm{erf}\;\Lambda -4\Lambda^{3}\left(1+\frac{4}{15}\Lambda^{2}\right)\mathrm{ierfc}\;\Lambda +\left(3+\frac{4}{3}\Lambda^{2}\right)\frac{2\Lambda }{5\sqrt{\pi }}e^{-\Lambda^{2}},$$

(74) $${\hat{Q}}_{\delta ,8}^{*}=\mathrm{erf}\;\Lambda -\frac{4}{5}\Lambda^{3}\left(3+\frac{8}{15}\Lambda^{2}\right)\mathrm{ierfc}\;\Lambda +\left(11+\frac{8}{3}\Lambda^{2}\right)\frac{2\Lambda }{25\sqrt{\pi }}e^{-\Lambda^{2}},$$

(75) $${\hat{Q}}_{\delta ,9}^{*}=\mathrm{erf}\;\Lambda +\frac{4}{5}\Lambda \left(3-\Lambda^{2}-\frac{8}{15}\Lambda^{4}\right)\mathrm{ierfc}\;\Lambda -\left(1-\frac{8}{15}\Lambda^{2}\right)\frac{2\Lambda }{5\sqrt{\pi }}e^{-\Lambda^{2}},$$

(76) $$\begin{array}{l} {\hat{Q}}_{\delta ,10}^{*}=4-3\mathrm{erf}\;\Lambda -2\left(3\sqrt{\pi }+6\Lambda +2\sqrt{\pi }\Lambda^{2}+2\Lambda^{3}\right)\mathrm{ierfc}\;\Lambda +\\ \;\;\;\;\;\;\;\;\;\;\;\;\;\;\;\;\;\;\;\;\;\;\;\;\;\;\;\;\;\;\;\;\;\;\;\;\;\;\;\;\;\;\;\;\;\;\;\;\;\;\;\;\;\;\;\;+\left(\sqrt{\pi }+\Lambda \right)\frac{2}{\sqrt{\pi }}e^{-\Lambda^{2}}. \end{array}$$

Moving in Formulas (66)–(76) to the limit δ→∞ (Λ→∞), the second of the Formulas (42) was written in the form:

(77) $${\hat{Q}}_{i}=\sqrt{kt_{s}}{\hat{Q}}_{i}^{*}\;,\;i=1,2,3,\ldots ,10,$$

where

(78) $${\hat{Q}}_{i}^{*}=1\;,\;i=1,2,4,\ldots ,10,$$

(79) $${\hat{Q}}_{3}^{*}=\frac{931}{896}\;≅1.039.$$

From Formulas (77) and (78), it follows that the parameters (41) of thermal saturation of the surface layer have the form

(80) $$Q_{\delta ,i}^{*}={\hat{Q}}_{\delta ,i}^{*},$$

where the parameters ${\hat{Q}}_{\delta ,i}^{*}$, i=1,2,4,…,10 are determined from Formulas (67), (68) and (70)–(77), respectively. In the case i=3, from Formulas (69) and (79) the following was obtained:

(81) $$\begin{array}{l} Q_{\delta ,3}^{*}=\mathrm{erf}\;\Lambda +\Lambda \left(\frac{840}{931}-\frac{840}{931}\Lambda^{2}-\frac{224}{2793}\Lambda^{4}-\frac{16}{4655}\Lambda^{6}\right)\mathrm{ierfc}\;\Lambda +\\ \;\;\;\;\;\;\;\;\;\;\;\;\;\;\;\;\;\;\;\;\;\;\;\;\;\;\;\;\;\;+\left(\frac{370}{931}+\frac{524}{13,965}\Lambda^{2}+\frac{8}{4655}\Lambda^{4}\right)\frac{\Lambda }{\sqrt{\pi }}e^{-\Lambda^{2}}. \end{array}$$

**Figure 2:**
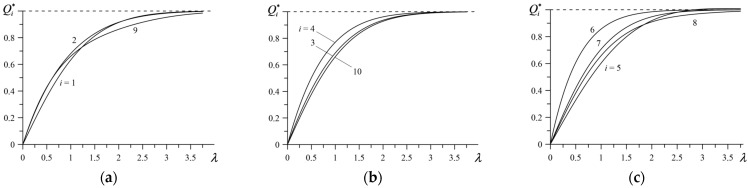
Change in the thermal saturation parameters $Q_{\delta ,i}^{*}$ (39) with increases in the coefficient λ: (**a**) i=1,2,9; (**b**) i=3,4,10; (**c**) i=5,6,7.

The dependencies of parameters $Q_{\delta ,i}^{*}$ (80), (81) on the coefficient λ are shown in Figure 2, and the corresponding numerical data are included in Table 2.

With the increase in the coefficient λ and, therefore, the thickness of the layer δ , the amount of heat accumulated in it increases (Figure 2). For example, the data contained in Table 2 show that during braking with constant deceleration (*i* = 1) the thickness of the layer in which 80% of the heat was accumulated can be determined using Formula (1) with λ=1.45. The thickest (λ=1.58) layer absorbing the same amount of heat is formed in the case of *i* = 9, and the thinnest (λ=0.84) is formed at *i* = 6.

## 5. Conclusions

Reliable estimation of effective heat penetration depths and thermal layer thicknesses plays a particularly important role in the design phase when determining the temperature mode of friction elements in braking systems. Based on appropriate analytical solutions for a semi-infinite body heated by a heat flux with a given temporal profile, an approach to estimating these parameters was proposed. Ten time profiles were considered, corresponding to the most general braking regimes. It was established that

- The ratio of the temperature at the effective depth to the temperature of the heated surface does not change with time. The same applies to the ratio of the amount of heat accumulated in the surface layer with effective thickness to the amount of heat absorbed by the entire body.
- The temporal profile of friction power density and, therefore, of heat flux intensity must be taken into account when determining the characteristics of the temperature mode of the braking system.
- Developing a methodology for determining the effective heat transfer depth is also important in developing analytical models of frictional heating during braking. This parameter serves as a reference parameter when transitioning to dimensionless variables (spatial and temporal).
- The proposed approach is quite universal in the sense that it can be easily extended to other friction systems such as layer–substrate, layer–layer, etc.

Finally, it should be noted that the solution of the boundary value problem of heat conductivity (8)–(10) at constant intensity for the heat flux intensity $\left(q_{0}^{*}(t)=1\right)$ is known, and has the following form [12]:

(82) $$\Theta_{0}(z,t_{s})=\frac{2q_{0}}{K}\sqrt{kt_{s}}\;\mathrm{ierfc}\;Z,\;\Theta_{0}(0,t_{s})=\frac{2q_{0}}{K}\sqrt{\frac{kt_{s}}{\pi }}\;.$$

Hence, based on definition (25), it follows that

(83) $$\Theta_{\delta ,0}^{*}=\sqrt{\pi }\;\mathrm{ierfc}\;\Lambda ,$$

where the parameter Λ has the form expressed in definition (26). Calculations performed using Formula (83) showed that the values λ=1.73 and λ=3.2 mentioned in the Introduction were obtained for the values $\Theta_{\delta ,0}^{*}=0.13$ and $\Theta_{\delta ,0}^{*}=0.01$, respectively. However, using them in the case of a heat flux with a time-varying profile of may cause errors in estimating the temperature mode of the braking system. The solution to this problem is presented in this article.

## Figures and Tables

**Table 1:** The values of the coefficient λ at different values of the parameters $\Theta_{\delta ,i}^{*}$.

	$\Theta_{i}^{*}$	0.9	0.8	0.7	0.6	0.5	0.4	0.3	0.2	0.1	0.05	0.01
*i*												
1		0.41	0.63	0.82	1.00	1.20	1.41	1.65	1.96	2.43	2.83	3.64
2		0.07	0.17	0.26	0.37	0.50	0.64	0.83	1.08	1.49	1.87	2.65
3		0.19	0.37	0.54	0.72	0.92	1.13	1.38	1.70	2.17	2.59	3.43
4		0.09	0.19	0.30	0.43	0.57	0.74	0.95	1.23	1.67	2.07	2.90
5		0.51	0.75	0.96	1.16	1.36	1.58	1.82	2.14	2.60	3.00	3.81
6		0.07	0.13	0.21	0.30	0.40	0.53	0.68	0.90	1.25	1.58	2.30
7		0.33	0.50	0.66	0.81	0.98	1.16	1.37	1.65	2.07	2.44	3.21
8		0.37	0.56	0.74	0.91	1.09	1.29	1.53	1.83	2.28	2.68	3.49
9		0.11	0.22	0.34	0.48	0.63	0.80	1.01	1.28	1.71	2.09	2.88
10		0.38	0.58	0.75	0.93	1.11	1.31	1.55	1.84	2.28	2.68	3.48

**Table 2:** Coefficients λ corresponding to selected values of the parameters $Q_{\delta ,i}^{*}$.

	$Q_{i}^{*}$	0.1	0.2	0.3	0.4	0.5	0.6	0.7	0.8	0.9
I *i*										
1		0.13	0.27	0.42	0.57	0.74	0.93	1.16	1.45	1.91
2		0.10	0.20	0.32	0.46	0.62	0.81	1.05	1.37	1.89
3		0.11	0.22	0.35	0.48	0.63	0.81	1.03	1.30	1.74
4		0.08	0.17	0.27	0.38	0.50	0.65	0.84	1.09	1.50
5		0.15	0.30	0.45	0.62	0.80	0.99	1.23	1.51	1.90
6		0.06	0.12	0.20	0.27	0.38	0.49	0.65	0.84	1.18
7		0.12	0.23	0.35	0.48	0.62	0.79	0.99	1.25	1.66
8		0.13	0.26	0.39	0.53	0.69	0.88	1.11	1.41	1.93
9		0.10	0.20	0.32	0.46	0.63	0.85	1.13	1.58	2.34
10		0.13	0.25	0.39	0.53	0.69	0.87	1.09	1.38	1.81

## Data Availability

The original contributions presented in this study are included in the article. Further inquiries can be directed to the corresponding author.

## Nomenclature

$A_{a}$	Area of the nominal contact region ${(m}^{2})$
c	Specific heat, (J kg^−1^ K^−1^)
f	Dimensionless coefficient of friction
k	Thermal diffusivity, (m^2^ s^−1^)
K	Thermal conductivity, (W m^−1^ K^−1^)
$p_{0}$	Nominal value of the contact pressure, (Pa)
q	Intensity of the heat flux ($W\;m^{-2}$)
$q_{0}$	Nominal value of the intensity of the heat flux ($W\;m^{-2}$)
Q	Amount of heat absorbed during braking (J)
t	Time (s)
$t_{s}$	Stopping time (s)
T	Temperature, (K)
$T_{0}$	Initial temperature, (K)
$V_{0}$	Initial speed, (m s^−1^)
z	Spatial coordinate in axial direction, (m)
δ	Effective thickness of the surface layer (m)
γ	Dimensionless heat partition ratio
λ	Dimensionless coefficient
Θ	Temperature rise (K)
ρ	Density (kg m^−3^)
**Superscript**	
^*^	Dimensionless quantities
